# Social versus independent interest in 'bird flu' and 'swine flu'

**DOI:** 10.1371/currents.RRN1036

**Published:** 2009-09-03

**Authors:** R. Alexander Bentley, Paul Ormerod

**Affiliations:** ^*^Anthropology Dept., Durham University and ^†^Volterra Consulting

## Abstract

The explosion of interest in H1N1, more popularly called ‘swine flu’, across the world, from late April to early May 2009, exemplified how information transmission in modern online society can affect the spread of the disease itself. A simple but effective model based on cultural evolutionary theory can characterise in such data the effective degree of social transmission versus independent decision. In a novel approach that applies this model to Google Trends search data, we find significant differences in social transmission of the exact phrase `swine flu' in 2009, compared with ‘bird flu’ in 2005. The methodology can thus inform policies for addressing public awareness of health issues, which can be more effective with knowledge of how the information is being spread or learned.

## Introduction

The explosion of interests in ‘bird flu’ during 2005, and ‘swine flu’ from late April to early May 2009, were remarkable demonstrations of rapid cultural evolution and transmission [1]. This hysteria affected behaviour in real ways (from xenophobia to the closing of schools), and exemplified the importance of cultural transmission in modern globalised society, where such transmission of functional information affects the spread of the disease itself [2].

These two cultural phenomena provide an valuable opportunity to study cultural evolution on the global population scale. Basic theory in epidemiology and evolutionary biology can provide useful tools [2] [3] [4] [5], but often these methods treat the idea itself almost purely as a spreading virus. 

We suggest that general approaches of cultural evolutionary theory [3] [6] [7] offer a better way of analysing interest in health issues such as influenza.  A simple, but highly effective, model can characterise the effective degree of social transmission (adopt the idea because other people have adopted it, regardless of what it is) versus independent decision (adopt it because of a conscious decision made independently by the individual). This simple spectrum provides a powerful means of evaluating forms of cultural transmission amongst humans [4] [8].

## Model

One established formulation (e.g. 8, 9) of this model represents the probability of adoption at time *t* as:

                                          

\begin{equation*}p(t) = (\mu + qF(t))(1-F(t)),\end{equation*}

                                           (1)

where the parameters *q *and* µ *represent the degree of social transmission and individual decision, respectively. The cumulative fraction of adopters in (1), *F*(*t*),  is given by [9]:

                                                     

\begin{equation*}F(t)=A\frac{1-e^{-(\mu+q)t} }{1+\frac{q}{\mu} e^{-(\mu+q)t} }\end{equation*},

                                            (2)

where *A *is the maximum cumulative fraction of adopters in the population.

At one extreme of this model, we assume that social transmission is much higher than independent selection, i.e. that *µ *<< *q*. This yields essentially the standard logistic growth or ‘S’ curve model, in which the rate of adoption increases initially, peaks, and then decreases as the cumulative number of adopters asymptotically approaches its pre-determined maximum, *A*.

The number of new additions per time unit is the partial derivative of *F*(*t*) with respect to *t, *which, when we assume that *µ *<< *q*, is:

     

\begin{equation*}\frac{\partial F}{\partial t} \approx A\frac{\left( 1+\frac{q}{\mu}e^{-qt} \right)qe^{-qt}+q\frac{q}{\mu}e^{-qt} -\frac{q^2}{\mu}e^{-2qt} }{\left( 1+\frac{q}{\mu}e^{-qt} \right)^2 }\end{equation*}

                                        (3)

which simplifies to:

                                   

\begin{equation*}\frac{\partial F}{\partial t} \approx A\frac{qe^{-qt}\left( 1+\frac{q}{\mu} \right) }{\left( 1+\frac{q}{\mu}e^{-qt} \right) ^2}\end{equation*}.

                                             (4)

Eq. 4 yields a symmetrical pattern of rise and decline in terms of new additions per time unit.

The other end of the spectrum has been explored theoretically [10], but rarely in real-world case studies. At this end, we assume that independent selection of the behaviour is much greater than social transmission, i.e., *µ *>> *q*, whereby eq. (2) becomes in the limit as *q* approaches zero :

                                               

\begin{equation*}F(t)\approx A(1-e^{-\mu t})\end{equation*},

                                                     (5)

with derivative:

                                       

\begin{equation*}\frac{\partial F}{\partial t} \approx A\mu e^{-\mu t}\end{equation*}.

                                                                      (6)

Hence with purely independent decisions, the adoption rate is most rapid initially, at *t* = 0, followed by a simple exponential decline in the rate of adoption.  This implies instantaneous rise to peak adoption rate i.e. very strong asymmetry in the rise and decline in terms of new additions per time unit.

## Data

To observe the relative level of global interest in recent pandemic scares, we make use of the new, powerful online analysis tool, “Google Trends”. This tool provides a calendar timeline of search results for any search term, which is sub-divisible by country (or even regions within each country). Google Trends does not provide the exact numeric data on the volume of searches, but a relative account of search volume over time.  The data are typical of much data in the social sciences, and cannot be regarded as having the precision of a replicable experiment in the natural sciences.  However, these are data to which Google are willing to attach their name, and as such can be regarded as a reliable indicator of movements over time.

For April-May 2009, we obtained search data (world-aggregated) for ‘swine flu’, and the  related topic ‘bird flu’. The period of growth and decline in both terms was defined to be 24 April – 4 May 2009. We also collected search data for ‘bird flu’ from 1 September to 30 December 2005, a period when it was a genuine threat.  The searches in April-May 2009 for ‘swine flu’ were so abundant that we also had the opportunity to compare the rise and fall of this term in 30 individual countries (which was not possible for ‘bird flu’, unfortunately).

## Analysis

For each time series of Google search volume – ‘swine flu’ in 2009 and ‘bird flu’ in 2005 and in 2009 – we found the best fit of these data to the model, represented by rate of change in eq. (2), from one ‘day’ (*t*)to the next (*t *+ 1). In this way, we obtained statistical estimates of the social transmission and independent decision parameters,* q *and *µ*, for time series data on each of the phrases aggregated across countries (Table 1).

**Table:** 

**Event** ** **	** Model fit (r** ^**2**^ **)**	** ** **μ**	** ** \begin{equation*}q\end{equation*}	** ** \begin{equation*}\frac{q}{\mu}\end{equation*}
**Swine flu '09**	** ** \begin{equation*}0.86\end{equation*}	** ** \begin{equation*}0.047\pm 0.028\end{equation*}	** ** \begin{equation*}0.59 \pm 0.16\end{equation*}	** ** \begin{equation*}12.6\end{equation*}
** Bird flu '09**	** ** \begin{equation*}0.75\end{equation*}	** ** \begin{equation*}0.049 \pm0.034\end{equation*}	** ** \begin{equation*}0.43 \pm 0.17\end{equation*}	** ** \begin{equation*}9.1\end{equation*}
** Bird flu '05, to 29 Oct**	** ** \begin{equation*}0.93\end{equation*}	** ** \begin{equation*}<0.001\end{equation*}	** ** \begin{equation*}0.137 \pm 0.013\end{equation*}	** ** \begin{equation*}> 100\end{equation*}
** Bird flu '05, after 29 Oct**	\begin{equation*}0.86\end{equation*}	\begin{equation*}0.043 \pm 0.007\end{equation*}	\begin{equation*}< 0.001\end{equation*}	\begin{equation*}0\end{equation*}

For the 2005 ‘bird flu’ phenomenon, we found there were clearly two distinct search successive events (Figure 1a).  The first built up to a peak on 17 October and then fell away steadily until 29 October. This was followed by a clear, rapid surge in searches for ‘bird flu’, peaking on 2 November and subsequently declining steadily to the end of the year. The distinction of these two events is robust; with the sample subdivided this way, regression versus the model yields pooled standard errors significantly lower than the model versus the whole period of data.

**Figure:**
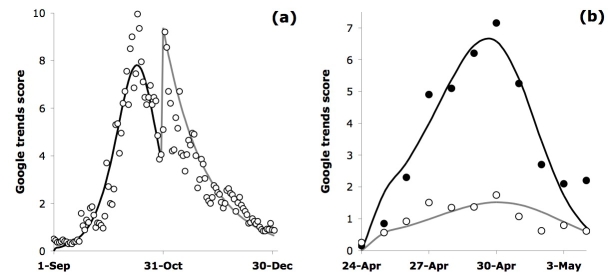
**Figure 1: (a) **Daily, world-aggregated Google volume for ‘bird flu’ in late 2005 (open symbols), versus a two-part model (parameters, Table 1); the second part starting 30 October. **(b) **Google Trends results, for ‘swine flu’ (filled symbols), and ‘bird flu’ (open symbols), for the period April-May 2009, versus the imitation-selection model tuned to each case (Table 1).

These two periods of ‘bird flu’ searches are not only distinct, but different in character.  In the initial period (up to 29 October 2005), the model fits best (*r^2^* = 0.93) with the independent decision parameter *µ* effectively at zero, and the social transmission parameter *q *at 0.137 ± 0.013 (error denotes 95% confidence interval).  Over the second period (after 29 October), however, the best overall fit (*r^2^* = 0.86) is achieved with social transmission parameter *q* effectively zero (Table 1), whereas the independent decision parameter *µ*  is 0.043 ± 0.007.  Figure 1a shows how the model fits the data using these quite different parameter values for the respective time periods.

For the more recent event, over the period 24 April to 4 May 2009, Figure 1b shows the aggregated result from all countries for both ‘swine flu’ and ‘bird flu’. For ‘swine flu’ the data are fitted reasonably well (*r^2^* = 0.86) by the model, with independent decision* µ* = 0.047 ± 0.028 and social transmission* q* = 0.592 ± 0.155.  For 'bird flu' 2009, the best fit (*r^2^* = 0.75) occurs with independent decision* µ* = 0.049 ± 0.034 and social transmission* q* = 0.428 ± 0.168.

For the 2009 event, the rise and fall of ‘swine flu’ in 30 different countries individually was observed by recording the date of four key landmarks: (1) initial appearance at a detectable level, (2) climbed to half its peak popularity (3) reached peak popularity, and (4) declined back to half of peak popularity.   Since the data are reported only on a daily basis, we approximated the ‘half peak’ date either by rounding to the nearest day, or, in cases where the half peak value was clearly spanned by two successive dates, 26 and 27 April say, we assigned the ‘date’ of ‘26.5 April’ to the relevant half peak.  More sophisticated interpolation could certainly be used, but the Google data are not generated by a controlled scientific experiment, and so should not be assigned undue precision.

An asymmetry in the rise-decline patterns across the countries is evidenced by an inverse correlation between the rising time (from half peak to the peak) versus decline time (from peak to half-peak level), shown in Figure 2. For these 30 different countries, the simple correlation is -0.74 (*r* ^2^ =  0.70), with an equation standard error of 0.751 and an optimal effective number of parameters of 2.4 using non-linear least-squares estimation [11].  The null hypothesis that the relationship is linear is rejected at *p* = 0.002.

**Figure:**
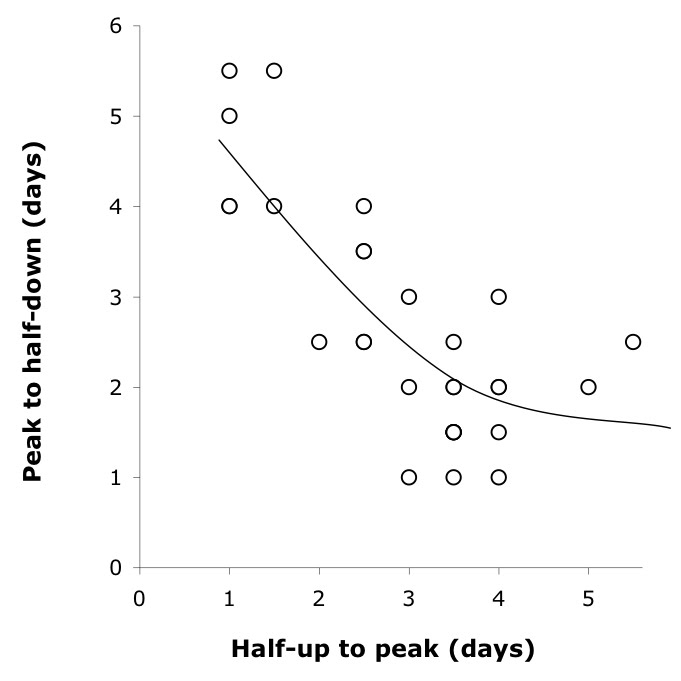
**Figure 2**:Rise/fall times of‘swine flu’ Internet search rates. Each datum represents a different country, showing the number of days between landmarks 2 and 3 (x-axis), versus days between landmarks 3 and 4 (y-axis). The nonlinear regression line is described in the text.

## Discussion

Fear is contagious [12], and fitting the model to the aggregated time series suggests social transmission was more important than independent decision in both the pandemic search cases in April/May 2009.  Nonetheless, the estimated *μ* values are between 4% and 5%, which is at the high end of the range of values for independent decision in comparable studies of idea diffusion (e.g., 8, 13).  Although 5% may not seem like much, studies of social learning in animals suggest such a minority of independent behaviour can lead an entire group in a cohesive direction [13].   

For the phrase ‘swine flu’, significant independent decision is also evidenced by an asymmetry between the speed of rise and speed of decline in the cross-country observations. Given also the relatively high *μ* value, this presumably reflects independent responses to the media portrayal of this strain of influenza. As defined by the time between Landmark 3 (half-down) and the peak, the 'swine flu' searches resonated longer in Asian countries, including Thailand, Singapore, Malaysia, China, the Philippines, and Japan, whereas the buzz had died down most thoroughly in European-American countries (Supplementary Table S1). Given the recent history of avian influenza in Asia, there is reasonable case for genuine concern about it in Asia.

In comparing the different case studies, a useful statistic may be the ratio *q/µ, *which characterises social transmission relative to independent decision. Table 2 shows that, during the 2009 event, the ‘swine flu’ had an social/independent ratio of 12.6, whereas for ‘bird flu’ was 9.1.  These are not substantial differences, but they nevertheless offer support to the hypothesis that the more distinct the related phenomena is from the main, socially-transmitted phenomenon, the more independent decision was involved.   In other words, people were socially copying each other’s interest in ‘swine flu’, but making the connection between this and ‘bird flu’ required knowledge, and therefore more independent selection. 

The 2005 ‘bird flu’ scare provides a remarkable demonstration of the extremes: there was almost pure imitation in September and most of October, followed by almost pure independent decision after 29 October.

Alongside the many studies of the actual viruses, evolutionary studies of concern over these health issues could have significant implications for informing the public, where optimal strategies could be quite different depending on whether decisions are made primarily socially, or primarily independently. If decisions are made independently, then informative news can have a powerful effect on people. If decisions are made mainly by social transmission, however, the provision of information in itself, no matter how good, is much less likely to be taken up. 

### Acknowledgment  

We thank Rich Colbaugh for valuable comments.

### Competing interests

The authors have declared that no competing interests exist.

#### Funding information

This research was supported by the Centre for the Coevolution of Biology and Culture, Durham University, UK.
